# Cytological patterns of thyroid lesions in Najran, Saudi Arabia: A 5-year retrospective study

**DOI:** 10.15537/smj.2022.43.7.20220223

**Published:** 2022-07

**Authors:** Saad M. Alqahtani

**Affiliations:** *From the Department of Pathology, College of Medicine, Najran University Hospital, Najran University, Najran, Kingdom of Saudi Arabia*.

**Keywords:** thyroid, FNAC, Saudi Arabia, cytopathology, Bethesda

## Abstract

**Objectives:**

To assess the cytological pattern of thyroid lesions in Najran, Saudi Arabia.

**Methods:**

A retrospective study, from the period of January 2015 to December 2019. All patients with thyroid enlargement who were presented to different hospitals in Najran and assessed by fine needle aspiration cytology were included in this study.

**Results:**

Of 1353 cases, 1138 (84.1%) were female and 215 (15.9%) were male. Most of the thyroid lesions were benign (72.5%) including follicular nodules (39.5%), Hashimoto’s disease (21.2%), and colloid nodules (11.8%). There were 107 (7.9%)cases of suspicious malignancy, and the most common malignant tumor was papillary carcinoma (10.2%). The 2^nd^ age group (21-40 years) was the common age to be diagnosed with malignant tumors, particularly in males. The 3^rd^ age group (41-60 years) was most affected by thyroid lesions, particularly in females.

**Conclusion:**

Most of thyroid lesions in Najran were benign, and females were more affected by thyroid lesions than males. However, papillary carcinoma was the 4^th^ most frequent thyroid lesion in females, while it was the 2^nd^ most frequent in males and diagnosed mainly in younger males (21-40 years). Finally, ages 21-60 years were associated with most of the thyroid lesions in both males and females.

**I**t is known that thyroid nodule is a common presentation, and fine needle aspiration cytology (FNAC) is considered its initial screening test.^1^ Fine needle aspiration cytology is a quick, inexpensive, highly diagnostic, and sensitive tool to classify thyroid nodules or lesions as either benign or malignant lesions.^2^ The great impact on advancement and improvement of healthcare due to the implementation of the thyroid FNAC has been illustrated in numerous studies that have compared the accuracy of FNAC with the final histopathology diagnosis.^3-5^ Overall, research has found that FNAC has a high diagnostic sensitivity and specificity levels. Additionally, patients with preoperative FNAC had an optimal surgical treatment, compared with patients who had no preoperative FNAC.^6^ Therefore, FNAC appears to be an important tool in planning the treatment procedure for thyroid nodules, avoiding unnecessary surgeries and improving surgical outcomes.

The Bethesda system for reporting thyroid cytopathology was presented in 2007 and was updated in 2017 to standardize reporting of thyroid FNAC and to minimize communication challenges between healthcare providers.^7^ The Bethesda system includes 6 categories for reporting thyroid cytology according to specific criteria. These categories are: I) non-diagnostic and unsatisfactory; II) benign; III) atypia of undetermined significance or follicular lesion of undetermined significance; IV) follicular neoplasm or suspicious for follicular neoplasm; V) suspicious for malignancy; and VI) malignant. Each category is assigned a certain level of risk of malignancy and recommends guidelines for clinical management. For instance, the benign lesion category is linked to a 3% risk of developing malignancy, and the recommended clinical management is a clinical follow-up. Additionally, suspicious malignant are linked to a risk of 60-75% of developing malignancy and malignant categories are linked to a risk of 97-99% of developing malignancy.^7^

In a descriptive study of thyroid malignancies in the Saudi population, thyroid cancer was found to be the second most common cancer among females and Najran had the highest differences rate which increased from 2001-2013.^8^ In a recent publication that examined the pattern of malignant tumors in Najran during 5 years, the thyroid gland was reported as the second most affected organ with a total of 130 (95 females and 35 males) cases.^9^ Furthermore, the pattern of thyroid lesions was studied in different regions across Saudi Arabia. For example, the most common thyroid disease in the Western region was the benign nodule, while papillary carcinoma was the most common neoplastic disease.^10^ Another study was carried out in Al-Madinah Al Munawarah, Saudi Arabia, and concluded that follicular adenoma was the most common benign tumor and papillary carcinoma was on the top of most common malignancies.^11^ The current study aims to assess the cytological pattern of thyroid lesions in Najran, according to the Bethesda system. This is because Najran has a high incidence of thyroid diseases in comparison to the other regions in Saudi Arabia, and the thyroid FNAC is considered an initial diagnostic tool in Najran.^8^

## Methods

The current study is a retrospective study to assess the cytological pattern of thyroid lesions in Najran, according to the Bethesda system. This study examined the thyroid FNAC received at the Departments of Pathology at Najran University Hospital, King Khalid Hospital, the General Hospital, and the Hospital of Maternity and Children, Najran, Saudi Arabia. The period of the assessment was from January 2015 to December 2019. All patients with thyroid enlargement who were assessed by FNAC were included in this study. Inclusion criteria included the presence of: I) detailed cytology report; II) single final diagnosis, and III) archived slides for each case. Any case did not meet the inclusion criteria was excluded. This research was approved by the local Ethics Committee at the College of Medicine, at Najran University, Najran, Saudi Arabia. The demographic data and diagnoses were collected from the archive of pathology reports considering Helsinki declaration while using human data. Thyroid diseases were classified according to their cytological features and categorized according to Bethesda diagnostic guidelines.

### Statistical analysis

Data were analyzed using the Prism Graph Pad 6 for Windows, version 6.07 (CA, USA). Categorical variables were analyzed using descriptive statistics, including frequency and percentages.

## Results

A total of 1353 cases were received from January 2015 to December 2019 at Najran hospitals’ Departments of Pathology. Of the 1353 cases, 1138 (84.1%) were females and 215 (15.9%) were males. There were 981 (72.5%) cases with benign lesions, 107 (7.9%) suspicious cases for malignancy, and 143 (10.6%) malignant cases.

**Figure 1:**
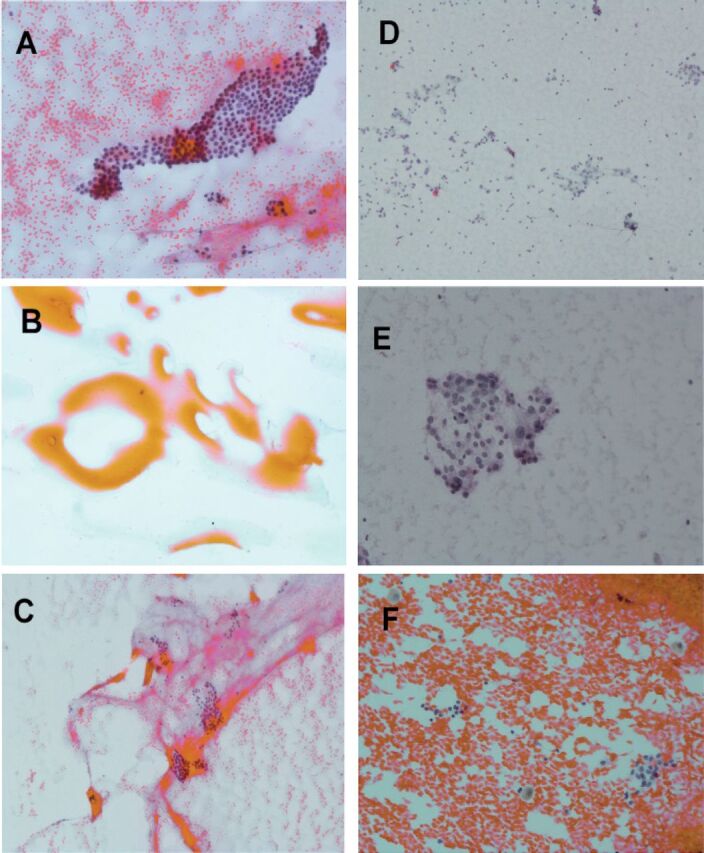
- Representative pictures for common benign thyroid lesions: A) 20X benign follicular cells forming monolayered macro-follicles; B) 20X Colloid lesion. C) 10X benign follicular cells with background of colloid; D) 10X Hashimoto’s (lymphocytic) thyroiditis; E) 20X Hashimoto’s (lymphocytic) thyroiditis; and F) 20X Pap stain shows benign follicular cells and hemosiderin laden macrophages.

**Figure 2:**
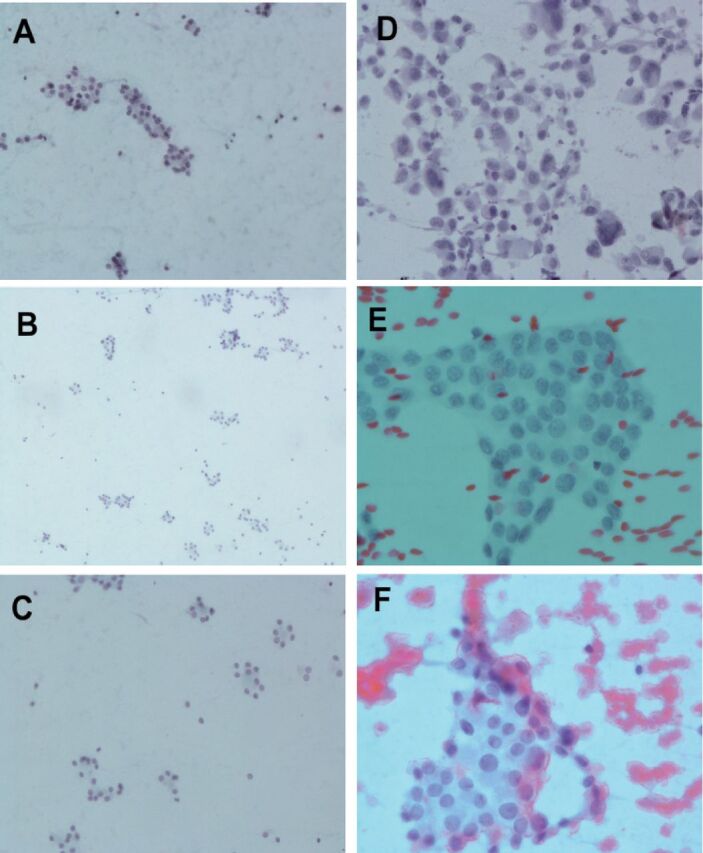
- Representative pictures for common malignant thyroid lesions: A) 20X Suspicious thyroid neoplasm; B) 10X Suspicious of follicular neoplasm; C) 20X Suspicious of follicular neoplasm; D) X20 anaplastic thyroid carcinoma E) 40X Pap stain papillary thyroid carcinoma, nuclear enlargement, irregular nuclear contour, and powdery chromatin and nuclear grooves; F) 40X papillary thyroid carcinoma with nuclear pseudoinclusion

According to Bethesda diagnostic criteria, cases were organized into 6 categories (Table 1). First, the nondiagnostic/unsatisfactory category had 76 (5.6%) cases diagnosed as inadequate material. The second category was benign lesions including: follicular nodule (39.5%), colloid nodule (11.8%), and Hashimoto’s thyroiditis also called lymphocytic thyroiditis (21.2%). Figure 1 shows representative pictures for the common benign lesions. The third category was the cell atypia of uncertain significance, and there were 32 (2.4%) cases signed as cell atypia. The fourth category was follicular neoplasms, suspicious follicular neoplasms, or Hürthle cell types; and it included follicular neoplasms (0.8%) and Hürthle cell type neoplasms (0.2%). The fifth category was suspicious for malignancy and included 107 (7.9%) cases. The sixth category was malignant neoplasms, and there were 138 (10.2%) cases of papillary carcinoma, and 5 (0.4%) cases of anaplastic carcinoma. Finally, Figure 2 includes representative pictures for the common malignant lesions.

**Table 1:** - The cytological pattern of thyroid lesions in Najran, Saudi Arabia according to Bethesda guidelines.

	**Bethesda diagnostic categories**	**Pathological diagnosis**	**n**	**%**
I	Non-diagnostic or unsatisfactory (76 cases, 5.6%)	Inadequate material	76	5.6
II	Benign (981, 72.5%)	Follicular nodule	535	39.5
		Colloid nodule	159	11.8
		Hashimoto’s thyroiditis	287	21.2
III	Atypia of undetermined significance or follicular lesion of undetermined significance (32, 2.4%)	Cell atypia	32	2.4
IV	Follicular neoplasms, suspicious follicular neoplasm, or Hürthle cell type (14, 1.0%)	Follicular neoplasms or suspicious follicular neoplasm	11	0.8
		Hürthle cell follicular neoplasm	3	0.2
V	Suspicious for malignancy (107, 7.9%)	Suspicious for malignancy	107	7.9
VI	Malignancy (143, 10.6%)	Papillary carcinoma	138	10.2
		Anaplastic carcinoma	5	0.4
		Total	1353	100

As the results show in Table 2, the most common thyroid lesion in females were as follows: follicular nodules (36.7%), Hashimoto’s thyroiditis (23.4%), colloid nodules (12.1%), papillary carcinoma (10%), suspicious for malignancy (7.8%), cell atypia (2.5%), follicular neoplasms/suspicious follicular neoplasms (0.9%), anaplastic carcinoma (0.4%), and Hürthle cell type neoplasms (0.2%). Meanwhile, the most common thyroid lesion in males were: follicular nodules (54.4%), papillary carcinoma (11.2%), colloid nodules (9.8%), Hashimoto’s thyroiditis (9.8%), suspicious for malignancy (8.4%), cell atypia (1.4%), follicular neoplasms/suspicious follicular neoplasms (0.5%), and Hürthle cell type neoplasms (0.5%).

**Table 2:** - The common thyroid lesions in male versus female according to cytology.

Pathological diagnosis	Total number of cases	Female			Male		
		Percentages	n	%	Percentages	n	%
Follicular nodule	535	39.5	418	36.7	39.45	117	54.4
Hashimoto’s thyroiditis	287	21.2	266	23.4	10.2	24	11.2
Colloid nodule	159	11.8	138	12.1	11.8	21	9.8
Papillary carcinoma	138	10.2	114	10.0	21.2	21	9.8
Suspicious for malignancy	107	7.9	89	7.8	7.9	18	8.4
Inadequate material	76	5.6	67	5.9	5.6	9	4.2
Cell atypia	32	2.4	29	2.5	2.4	3	1.4
Follicular neoplasms or suspicious follicular neoplasm	11	0.8	10	0.9	0.8	1	0.5
Anaplastic carcinoma	5	0.4	5	0.4	0.2	1	0.5
Hürthle cell follicular neoplasm	3	0.2	2	0.2	0.4	0	0.0
Total			1138	100		215	100

The age distribution of the common thyroid lesions was assessed in females and males, and the patients were categorized in 4 groups. Group 1 included patients who are 20 years old or younger, Group 2 for patients aged 21-40, Group 3 for those aged 41-60 years, and Group 4 for patients more than 60 years old. The most frequently affected female patients (Table 3) with thyroid lesions were age Group 3 (55.9%), and the females aged 21-40 years in Group 2 were in the second position (32.1%). The females in age Group 4 who are older than 60 years old were the third age group reported with thyroid lesions (11.4%), and only 7 (0.6%) patients of the diagnosed females with thyroid lesions were younger than 20 years old. Additionally, papillary carcinoma; follicular neoplasms and suspicious cases for malignancy were diagnosed mainly in age Group 3 (41-60 years). However, anaplastic carcinoma was only diagnosed in age Group 2.

**Table 3:** - Age distribution of the common thyroid lesions in females.

Pathological diagnosis	Total number of cases	Female	Group 1 0-20 Y	Group 2 21-40 Y	Group 3 41-60 Y	Group 4 >60 Y
Follicular nodule	535	418	0	92	251	75
Hashimoto’s thyroiditis	287	266	0	139	115	12
Colloid nodule	159	138	4	61	69	4
Papillary carcinoma	138	114	0	29	83	2
Suspicious for malignancy	107	89	0	11	71	7
Inadequate material	76	67	3	17	18	29
Cell atypia	32	29	0	9	20	0
Follicular neoplasms or suspicious follicular neoplasm	11	10	0	2	7	1
Anaplastic carcinoma	5	5	0	5	0	0
Hürthle cell follicular neoplasm	3	2	0	0	2	0
Total	1353	1138	7	365	636	130
Percentage	0.6	32.1	55.9	11.4		

Y: years

On the other hand, the most frequently affected males (Table 4) with thyroid lesions was age Group 2 (53.5%), then age Group 3 (28.4%), Group age 4 (17.7%), and only one (0.5%) case in age Group 1 as the least frequent age group. Papillary carcinoma was diagnosed in 24 males and mainly diagnosed in age Group 2 (16 cases) and age Group 4 (7 cases). Finally, Hashimoto’s thyroiditis was mainly in age Group 3.

**Table 4:** - Age distribution of the common thyroid lesions in males.

Pathological diagnosis	Total number of cases	Male	Group 1 0-20 Y	Group 2 21-40 Y	Group 3 41-60 Y	Group 4 >60 Y
Follicular nodule	535	117	0	67	31	19
Papillary carcinoma	138	24	0	16	1	7
Colloid nodule	159	21	0	15	6	0
Hashimoto’s thyroiditis	287	21	0	4	16	1
Suspicious for malignancy	107	18	0	9	3	6
Inadequate material	76	9	1	1	2	5
Cell atypia	32	3	0	3	0	0
Follicular neoplasms or suspicious follicular neoplasm	11	1	0	0	1	0
Hürthle cell follicular neoplasm	3	1	0	0	1	0
Anaplastic Carcinoma	5	0	0	0	0	0
Total	1353	215	1	115	61	38
Percentage			0.5	53.5	28.4	17.7

Y: year

## Discussion

Thyroid nodules are common lesions that are single or multiple within the gland. These lesions are commonly found among females, compared to males.^6,12^ This is consistent with the results of the current study, as there were 1138 (84.1%) females out of 1353 cases diagnosed with thyroid lesions. However, comparing the most common thyroid diseases in males to females, papillary carcinoma was the second most common disease in males (11.2%) while it was the fourth common disease in females (10%). The latter observation has been reported previously in different studies that found that males with thyroid nodules displayed a higher incidence of malignancy than females.^13,14^

The vast majority of thyroid nodules are known to be benign, while only up to 15% are malignant.^7^ Results from the current study showed that most of the thyroid lesions in Najran were benign (72.5%). The benign lesions were follicular nodules (39.5%), colloid nodules (11.8%), and Hashimoto’s thyroiditis (21.2%). In both females and males, the benign category of Bethesda system was the most common diagnosis. In females, follicular nodules (36.7%), Hashimoto’s thyroiditis (23.4%), and colloid nodules (12.1%) were the most common benign lesions. In males, the follicular nodules (54.4%), colloid nodules (9.8%), and Hashimoto’s thyroiditis (9.8%) were the most common benign lesions. These results are in concurrence with the literature, as benign thyroid lesions are very commonly reported compared to malignant lesions.^7,15^ In addition, it has been reported that females are affected by Hashimoto’s disease more often than males, and the results of this study showed that Hashimoto’s disease was more frequent in females (23.4%) than males (9.8%).^16^

There were 107 suspicious malignant cases and 143 malignant thyroid tumors. The malignant tumors were mainly papillary carcinoma in 138 cases and only 5 cases of anaplastic carcinoma. This observation is supported by a study that analyzed 600 cases from tertiary hospitals in Saudi Arabia and found that more than 77% of the malignant thyroid tumors were papillary carcinoma.^15^ Additionally, a recent study in Najran reported papillary thyroid carcinoma as the second common carcinoma after adenocarcinoma.^9^ Similarly, 2 studies in the western region and Al-Madinah Al-Munawarah region of Saudi Arabia reported that papillary carcinoma was on the top of most common malignant tumor.^10,11^ Interestingly, previous studies have reported other malignant thyroid tumors besides papillary carcinoma and anaplastic carcinoma, but in the current study there was no medullary carcinoma or lymphoma found.^10,15^

The size and number of nodules were more closely linked to development of malignancy than age.^13,17^ Moreover, some reports have revealed no significant relation between increased age as a single parameter and the development of thyroid malignancy.^18,19^ However, some publications have concluded a positive significant relation between increased age and the development of thyroid cancer. In the current report, the most frequently affected female patients with thyroid diseases (55.9%) were from age Group 3 (41-60 year-old) than age Group 2 (21-40 year-old). However, males in age Group 2 (21-40 year-old) had an age most associated with thyroid lesions (53.5%) compared to age Group 3 (28.4%). Collectively, the age period of 21-60 years in both females and males was associated with most of the thyroid lesions. Noteworthy, malignant tumors were diagnosed mainly in patients younger than 60 and older than 20 year-old in females and males. The latter observation opposes linking old age as a single parameter to the development of thyroid cancer.^18,19^ In contrast, this observation is consistent with other reports from Saudi Arabia that have concluded that the third and fourth decades of life are most commonly affected by thyroid lesions.^10,11^

### Study limitations

The unavailability of histopathology and follow up since most of the patients seek the treatment in central and specialized hospitals outside Najran. Another limitation is the exclusion of 209 cases that did not meet the inclusion criteria.

In conclusion, the results of this study are in concurrence with similar studies carried out in different regions of Saudi Arabia. Most of the thyroid lesions in Najran were benign, and females were affected by thyroid diseases more often than males. Papillary carcinoma was the fourth most frequent thyroid lesion in females, while it was the second most frequent in males, particularly younger males (21-40 years). The age period of 21-60 years in both females and males was associated most with thyroid lesions development, and patients younger than 20 or older than 60 years were associated with the least chance to develop benign or malignant thyroid lesions. Finally, this study may be useful as a background for further epidemiological or molecular studies and provides a valuable reference for the region’s pathologists.
